# Impact on and use of an inner-city London Infectious Diseases Department by international migrants: a questionnaire survey

**DOI:** 10.1186/1472-6963-7-113

**Published:** 2007-07-20

**Authors:** Graham Cooke, Sally Hargreaves, Jana Natkunarajah, Gurjinder Sandhu, Devesh Dhasmana, Joseph Eliahoo, Alison Holmes, Jon S Friedland

**Affiliations:** 1 International Health Unit, Department of Infectious Diseases and Immunity, Faculty of Medicine, Imperial College, Hammersmith Hospital Campus, London, W12 ONN, UK; 2 Statistical Advisory Service, Imperial College, London, SW7 2AZ, UK

## Abstract

**Background:**

The UK has witnessed a considerable increase in immigration in the past decade. Migrant may face barriers to accessing appropriate health care on arrival and the current focus on screening certain migrants for tuberculosis on arrival is considered inadequate. We assessed the implications for an inner-city London Infectious Diseases Department in a high migrant area.

**Methods:**

We administered an anonymous 20-point questionnaire survey to all admitted patients during a 6 week period. Questions related to sociodemographic characteristics and clinical presentation. Analysis was by migration status (UK born *vs *overseas born).

**Results:**

111 of 133 patients completed the survey (response rate 83.4%). 58 (52.2%) were born in the UK; 53 (47.7%) of the cohort were overseas born. Overseas-born were over-represented in comparison to Census data for this survey site (47.7% *vs *33.6%; proportional difference 0.142 [95% CI 0.049–0.235]; p = 0.002): overseas born reported 33 different countries of birth, most (73.6%) of whom arrived in the UK pre-1975 and self-reported their nationality as British. A smaller number (26.4%) were new migrants to the UK (≤10 years), mostly refugees/asylum seekers. Overseas-born patients presented with a broad range and more severe spectrum of infections, differing from the UK-born population, resulting in two deaths in this group only. Presentation with a primary infection was associated with refugee/asylum status (n = 8; OR 6.35 [95% CI 1.28–31.50]; p = 0.023), being a new migrant (12; 10.62 [2.24–50.23]; p = 0.003), and being overseas born (31; 3.69 [1.67–8.18]; p = 0.001). Not having registered with a primary-care physician was associated with being overseas born, being a refugee/asylum seeker, being a new migrant, not having English as a first language, and being in the UK for ≤5 years. No significant differences were found between groups in terms of duration of illness prior to presentation or duration of hospitalisation (mean 11.74 days [SD 12.69]).

**Conclusion:**

Migrants presented with a range of more severe infections, which suggests they face barriers to accessing appropriate health care and screening both on arrival and once settled through primary care services. A more organised and holistic approach to migrant health care is required.

## Background

International migrants are a diverse group, including students, migrant workers and their families, refugees, and more settled migrants. In recent years European countries, specifically the UK, have experienced a substantial increase in migrants arriving from resource-poor countries [1]; countries which may have poorly-developed health-care systems and differing patterns of infectious disease and ill-health. The consequences for the health systems of richer nations of increasing migration from resource-poor countries are growing in importance [2]. Infectious diseases may not be present on arrival and may surface as a result of incomplete vaccinations [3,4]. Health needs may be compounded by the high levels of social and economic deprivation faced on arrival to a new country and by the migration process itself [5].

European countries, including the UK, focus efforts on screening immigrants for tuberculosis on arrival if the individual is migrating from an area of high prevalence [6]. Screening and follow-up of newly arrived migrants by UK Port Health control units has been considered inadequate and ineffective [7] and there have been calls to rethink this approach. In other European countries models in-country to deliver screening and preventive health care to migrants are also considered insufficient [8]. Evidence-based screening protocols for these populations are lacking.

New arrivals to the UK are encouraged to register with community General Practitioners (GPs) for primary health services, which are free of charge to migrants here for a settled purpose. GPs act as gatekeepers to the full range of services available through the National Health Service (NHS). NHS services, particularly in high migrant areas, may have services in place to help facilitate access to GP services by newcomers to the Borough. However, provision is limited and it is unclear as to how effective such systems are. In addition, they may not consider the wide range of migrants entering the UK. We and others have previously found that GPs rarely provide formal screening for new arrivals: few asked about vaccination status, considered malaria or parasitic infection, or referred for formal tuberculosis screening [9]. Little is known about the approach taken by GPs towards migrants who stay in the country for longer periods, or those who make frequent visits to their countries of origin. Screening rates are known to be low among UK asylum seekers [10] and ethnicity more broadly is associated with inequalities in access at the service provider level, in terms of referral, screening, and treatment [11]. Migrant groups may face barriers to accessing appropriate and timely primary health care in-country both on arrival and once settled, which has been associated with language and communications barriers, legislative barriers, and lack of knowledge of the local health-care system [12-14]. These barriers may differ across different categories of migrants, for example entitlement to received free NHS care will differ between migrants in the UK on a work permit or those who are irregular migrants.

Shortfalls in data collection at UK health services means that migration status is not routinely documented. Little is therefore known about the implications of barriers to community health care and screening in terms of presentation of migrants at secondary services such as Infectious Diseases Departments, with a view to informing service delivery. The aims of this study were to investigate the impact on and use of an Infectious Diseases Department by migrants in a high migrant area.

## Methods

### Data collection

The study took place at an Infectious Diseases Department in an inner-city London teaching hospital in Hammersmith and Fulham, an area with a substantial migrant population and established ethnic minority communities [15]. The survey was carried out for 6 weeks during a 24 hour period; all NHS patients referred to the service and who were in-patients were asked if they would be willing to take part in the study. Staff stated clearly to patients that the survey was both voluntary and confidential and that all data would remain anonymous. The hospital translation team was on hand to describe the aims of the study to any non-English speaking patients, or patients who requested an interpreter, to support clinical staff with administration of the questionnaire survey and/or to support completion of the survey forms. The study was approved by the Hammersmith Hospital Research Ethics Committee.

### The questionnaire

The questionnaire survey form comprised 20 structured questions (6 open ended and 14 closed questions). The questionnaire is an adaptation of that used in a previous published study of migrants presenting to an Accident and Emergency Department (A&E Department or an Emergency room) [16]. Questions related to the patients place of birth, year of arrival to the UK, nationality, prior use of GP services, mode of referral, duration of illness prior to presentation (≤1 week, 1 week to 1 month, 1 month to 6 months, and >6 months), and immigration status (whether an asylum seeker or refugee), as well as questions exploring factors that may impact on the ability of the patient to access mainstream health services such as language ability. Staff were asked to write the patients unique hospital identifier on the top of each survey form before presenting it to patients; ethics approval was granted to follow up patients' clinical data where necessary.

### Data analysis

Patients were placed into two main analysis groups: UK born and overseas born. Overseas born was defined as being born in a country outside of the British Isles – The United Kingdom of Great Britain and Northern Ireland and the Republic of Ireland. In addition, we explored the sociodemographic characteristics of new migrants (≤10 years), settled migrants (>10 years), and migrants reporting refugee and/or asylum status, because these were identified as dominant groups in the dataset at the outset.

We compared differences between groups using two-way tables by Chi-Square or Fisher's exact test. We used logistic regression modelling to explore associations of explanatory variables and outcomes. All data were analysed in SPSS (version 12.0, SPSS Inc).

## Results

### Characteristics of presenting patients

111 of 133 presenting patients completed the survey providing a response rate of 83.4%. 58 (52.2%) of patients were born in the UK; 53 (47.7%) of the cohort were overseas born (Table 1). Overseas born patients were over-represented in comparison to local Census data for this survey site. 53 (47.7%) reported that they were born overseas, significantly higher than local 2001 Census data [15], which indicates a 33.6% overseas-born population in the Borough of Hammersmith and Fulham (proportional difference 0.142 [95% CI 0.049–0.235]; p = 0.002).

**Table 1:** Characteristics of patients at the Infectious Diseases Department

**Characteristics**	**Overall**	**UK born**	**Overseas born**
**Total n (%)**	111 (100%)	58 (100%)	53 (100%)
**Average age (years, SD)**	59.3 (SD 21.3)	62.8 (SD 21.7)	54.9 (SD 20.9)
**Sex (Male n [%])**	61 (54.9%)	28 (48.2%)	33 (62.3%)
**English as a first language**	77 (69.4%)	57 (98.3%)	20 (37.7%)
**Permanent registration with a GP**	99 (89.2%)	56 (96.6%)	43 (81.1%)
**Prior contact with a GP about current illness**	28 (25.2%)	16 (27.6%)	12 (22.6%)
**Referral via GP to hospital**	24 (21.6%)	12 (20.7%)	12 (23.1%)
**Admission through A&E**	71 (63.9%)	39 (67.2%)	32 (60.4%)
**Duration of hospitalisation >14 days**	60 (54.1%)	30 (51.7%)	30 (56.6%)
**Diagnosis of a primary infection**	47 (42.3%)	16 (27.6%)	31 (58.5%)
**Time in the UK** **≤5 years**	11 (9.9%)	NA	11 (20.7%)
**6–10 years**	3 (2.70%)	NA	3 (5.7%)
**>10 years**	39 (35.1%)	NA	39 (73.6%)

NA = Not applicable. SD = Standard deviation

Among the 53 overseas born, 14 (26.4%) were new migrants who had been in the UK 10 years or less, and 39 (73.6%) were settled migrants. Among the settled migrants, the majority (64.1%) had arrived in the UK pre-1975. 7 (50%) of new migrants reported their country of origin as African. The immigration status' of the new migrants comprised: 8 refugees/asylum seeker, 1 student, 2 working permits, 1 tourist, 2 unknown. 10 (18.9%) of 53 reported an immigration status of refugee or asylum seeker, of whom 6 had full refugee status, 2 were asylum seekers awaiting a decision, and 1 was a failed asylum seeker.

33 different countries of birth were reported in the overseas-born patients, from the following regions: Asia 16 (14.4%) of 111, Africa 11 (9.9), Caribbean 5 (4.5), South America 2 (1.8), Middle East 4 (3.6), Oceania 2 (1.8); North America 2 (1.8), Europe 11 (9.9). However, in terms of self-reported nationality, the majority (41 [77.4%] of 53) of overseas born patients self-reported their nationality as British. In addition, 3 (5.7%) reported their nationality as Somalian, and 1 (1.9%) of each of the following: British/Chinese, British/Jamaican, Australian, Ethiopian, Ghanaian, Mongolian, Sri Lankan, Ukrainian, and American.

### GP registration and mode of referral

99 (89.2%) of this cohort reported permanent registration with a GP. Chi-square or Fisher's exact test was used to explore associations between having a GP and other categorical variables. Not having a GP was strongly associated with being overseas born (p = 0.009), being a refugee/asylum seeker (p < 0.0001), being a new migrant (p < 0.0001), not having English as a first language (p = 0.007), and being in the UK for ≤5 years (p < 0.0001). One patient was a tourist from the USA and wouldn't have been expected to be registered with a GP.

Patients presented to the service in one of two ways: 71 (64.0%) patients were unselected general medical patients presenting to the hospital's A&E Department, whilst 40 (36.0%) were internal referrals for specialist advice from within other departments in the hospital. Patients referred to this Infectious Diseases service, which is well established, were either diagnosed as having an infection by the admitting medical or other specialist teams or had symptoms documented by other specialist teams or by GPs which suggested that further investigation for infection is warranted. Of those coming via the A&E Department only 24 (21.6%) of the cohort had been directly referred by their GP to this service, with no significant differences found across analysis groups.

### Clinical presentation

There were important clinical differences between the UK-born and the overseas born patients in this cohort. There were 2 deaths in the cohort, both in the overseas-born group, and both from sepsis. Four cases of tuberculosis (2 pulmonary, 1 CNS, and 1 lymph-node infection) were diagnosed in the overseas-born group and none in the UK-born. 3 of the 4 tuberculosis cases occurred among those reporting having ever been a refugee or asylum seeker.

31 (58.5%) of the overseas born had a primary diagnosis of an infection, compared to 16 (27.6%) of the UK-born. These patients presented to the hospital with an infectious disease as their primary diagnosis, for example tuberculosis, as opposed to for example, acquiring an in-hospital infection following presentation with another disease. 16 (27.6%) UK-born patients, presented with pneumonias/chest infections and/or urinary-tract infections (UTIs). Among the overseas born patients a very broad range of diseases were noted: sepsis (2 patients); endocarditis (2); gastroenteritis (2); hepatitis (1); cellulitis (1); pancreatitis (1); pyometritis (1); empyema (1); tuberculosis (2 pulmonary, 1 central nervous system, and 1 lymph-node infection); non-tuberculous chest infections (4), UTIs (2), sexually transmitted infection (1); HIV seroconversion illness (1). Other overseas born patients who presented with a primary diagnosis of an infection were recorded as: Lymes disease (1); wound infection (5); and fever (2).

### Infection as a primary diagnosis and duration of illness

47 (42.3%) of patients in this cohort presented with an infection as a primary diagnosis. Logistic regression was used to explore factors associated with infection as a primary diagnosis. We found a range of factors associated with presentation to this service with a primary infection, including refugee/asylum status, being male, being a new migrant, and being overseas born (Table 2). In addition we found that 10 of 11 overseas-born patients reporting arrival in the UK of = 5 years presenting with primarily infectious problems, including 3 patients with tuberculosis and one of each with endocarditis, infectious hepatitis, Lymes disease, HIV seroconversion, chronic hepatitis-B-associated cirrhosis, gastroenteritis, and self-limiting viral illness.

**Table 2:** Univariate analysis of factors associated with presentation with a primary infection

**Variable**	**Primary infection N (%)**	**Odds Ratio (95% CI)**	**p value**
**Sex**			
Female	16 (34.0)	1	p = 0.047
Male	31 (66.0)	2.19 (1.00–4.77)	
**English as first language**			p = 0.007
Yes	21 (44.7)	1	
No	26 (55.3)	3.16 (1.37–7.32)	
**Admission route**			p < 0.0001
A&E	18 (38.3%)	1	
Internal referral	29 (61.7%)	7.76 (3.23–18.6)	
**Duration of hospitalisation**			p = 0.032
≤14 days	16 (34.0)	1	
>14 days	31 (66.0)	2.33 (1.07–5.09)	
**UK born**			p = 0.001
Yes	16 (34.0)	1	
No	31 (66.0)	3.69 (1.67–8.18)	
**New migrant**			p = 0.003
No	35 (74.5)	1	
Yes	12 (25.5)	10.62 (2.24–50.23)	
**Refugee/asylum seeker**			p = 0.023
No	39 (83.0)	1	
Yes	8 (17.0)	6.35 (1.28–31.50)	

No significant differences were found between groups in terms of duration of illness prior to presentation. The majority of patients had had their condition for ≤1 week (58 [52.3%]), with 35 (31.5%) reporting duration of illness of 1 week to 1 month; 12 (10.8%) 1 month to 6 months, and 6 (5.4%) >6 months. Mean duration of hospitalisation was 11.74 days (SD 12.69) with no significant differences between groups in terms of time in hospital.

## Discussion and conclusion

This is one of the first attempts to collect hospital infectious diseases data relating to the migration status of presenting patients in the UK context. We found that overseas-born patients were a diverse group that presented in considerable numbers to this Infectious Diseases Department, and were over-represented in comparison to local estimates. The majority of overseas-born patients (73.6%) were settled migrants reporting British nationality, most of whom had been in the UK for considerable periods of time (64.1% pre-1975). A lesser number of the overseas-born patients were new migrants (26.4%), over half of whom were individual reporting refugee or asylum seeker status. Migrants were a diverse group that presented with a broad range and more severe spectrum of infections, differing from the UK-born population. Our findings suggest that migrants may not access appropriate health care either by comprehensive screening on arrival or through local primary health services once settled. This has potentially adverse consequences for health outcomes and hospital-based services.

In the absence of routine data collection at general Infectious Diseases Departments and other health services in the UK, this study sought to provide data on the presentation of migrants at an inner-city health service, and to explore implications for service providers dealing with this patient group. These data therefore cannot be considered generalisable to any particular migrant group in the UK. However, our findings will likely be generalisable to other health-care services in high migrant areas, in terms of the diverse range of presenting migrants and the differing disease patterns between the overseas born and UK-born population. In addition, these data highlight the problems that some migrant groups may experience in terms of accessing statutory health care in the UK.

These findings support those of studies from other European countries that routinely monitor migration status at services, in terms of the additional impact of migrants presenting with a diverse range of presenting diseases [8], and compare to our previous study of migrants attending a London-based A&E Department near this survey site [16]. The population of overseas-born patients identified in this cohort was dominated by more settled migrants who reported similar GP registration rates to the UK-born population. The predominant presence of migrants at the infectious disease service even after several years of living in the UK may be explained by the additional barriers that have previously been described at the service-provider level in terms of access to appropriate quality care and screening [10]. Additionally, their over-representation at this service may be associated with their poor socioeconomic status, their increased risk of developing infectious diseases some years after arrival, and/or return visits to their country or origin [2,17-20]. One US study, for example, found that 23% of overseas-born patients with tuberculosis in one county had travelled to a tuberculosis-endemic area within the preceding 2 years [17]; a similar phenomenon has been described among migrants with tuberculosis and HIV in the UK [18-20].

GP services are the perhaps the most appropriate place for screening [21]; many researchers have called for re-evaluation of current policies towards migrants in the UK and more research to evaluate new systems that seek to strengthen the community-based approach with a view to improving health outcomes. The dominant mode of referral by patients in our study was self-referral through the A&E Department; this supports findings from one other study that reported most patients with tuberculosis at one London hospital being admitted acutely ill via self-presentation to the A&E Department, most previously undiagnosed [22]. The authors report that the utilisation of expensive in-patient resources has significant implications for purchasers and providers of care in socioeconomically deprived areas. These issues may be compounded by recent policy shifts to tighten up on the identification and charging of a range of migrants for health care in the UK at hospitals [23], with a proposed expansion into GP and community-based services [24]. It is as yet unclear the extent to which migrants, including irregular migrants and failed asylum seekers, will be excluded from both preventive health care and screening if they are unable to register with a GP and/or cannot afford private patient fees, and the implications this will pose to secondary services such as Infectious Diseases Departments. Such policies may have a knock-on effect on access to services for settled migrants, who may be entitled to access health care. In addition, the current focus of the UK Port of Arrival Scheme on tuberculosis screening among new asylum seekers misses the increasing numbers of incoming migrants from resource-poor countries entering to work and study, who may also be at increased risk [1].

GP registration rates were generally high among presenting patients to this service, although among the refugees/asylum seekers only 40% attending this service reported having permanent GP registration. We found no association in this study between lack of GP registration, immigration status, and delays to accessing health care. Measuring whether patients have delayed accessing health care is difficult to do in light of the diverse number of variables involved, including the urgency of the clinical condition, so we urge caution in the interpretation of these findings. Delays to care among migrants has been described in other contexts and associated with inadequate provision of health services [25]. Ideally we would have collected more data on patients' previous contact with primary care and screening services; however, patient surveys of this kind must be manageable in order to generate a high response rate and the survey needed to be short and concise. In order to meet the needs of migrants, permanent registration with community GPs is necessary so that individuals access the full range of health services. Primary care physicians and physicians in A&E Departments and Infectious Disease Departments must be aware that both recently arrived and more settled migrants may present with a broad range of infections. Some physicians may require specific training and additional resources in areas with substantial migrant populations. A recent report from the UK's Health Protection Agency has called for targeted public-health action in the UK, including the provision of NHS services that reflect the needs of the affected populations, and increased surveillance and recording of migration status at NHS services with a view to improving health-care provision to this group [26].

The quality and appropriateness of health provision available through primary health services for migrants needs to be further explored and adapted to ensure prompt detection and treatment. The adoption of a more organised and holistic approach to migrant health care by primary-care providers, and the facilitation of improved access to such services, will ensure treatment of infectious problems present both on arrival and those that remain more common several years after arrival.

## Competing interests

The author(s) declare that they have no competing interests.

## Authors' contributions

All authors were involved in the study design, and in the acquisition and interpretation of data. GC, JE, and SH performed the statistical analysis. SH and JSF drafted the final article and all authors were involved in revising it critically for important intellectual content. All authors read and approved the final manuscript.

## Pre-publication history

The pre-publication history for this paper can be accessed here:


